# The Dosage of the Derivative of* Clostridium Ghonii* (DCG) Spores Dictates Whether an IFN*γ*/IL-9 or a Strong IFN*γ* Response Is Elicited in TC-1 Tumour Bearing Mice

**DOI:** 10.1155/2019/1395138

**Published:** 2019-04-28

**Authors:** Guoying Ni, Yong Wang, David Good, Jianwei Yuan, Xuan Pan, Jou Wei, Xiaosong Liu, Ming Q. Wei

**Affiliations:** ^1^The First Affiliated Hospital (Clinical Medical School), Guangdong Pharmaceutical University, Guangzhou, Guangdong 528458, China; ^2^School of Medical Science and Menzies Health Institute Queensland, Griffith University, Gold Coast, QLD 4333, Australia; ^3^Shandong Xin Wei Biopharma Co, Jinan, Shandong 250101, China; ^4^School of Physiotherapy, Australian Catholic University, Banyo, QLD 4014, Australia; ^5^Queensland Health, Bundaberg Hospital, Bourbong Street, Bundaberg, QLD 4670, Australia; ^6^Cancer Research Institute, The First People's Hospital of Foshan, Foshan, Guangdong 528000, China; ^7^Inflammation and Healing Research Cluster, University of the Sunshine Coast, Maroochydore DC, QLD 4558, Australia

## Abstract

**Background:**

Anaerobic* Clostridial* spores (CG) cause significant oncolysis in hypoxic tumour microenvironment and result in tumour regression in both animal models and clinical trials. The immune mediated response plays a critical role in the antitumour effect by the anaerobic spore treatment.

**Method:**

Human papillomavirus 16 E6/E7 transformed TC-1 tumour bearing mice were intravenously administered with low (1 × 10^8^ CFU/kg) or high dosage (3 × 10^8^ CFU/kg) of Derivative* Clostridial *spore (DCG).

**Results:**

Intravenous administration of the derivative of* Clostridial ghonii* (DCG) spores leads to both tumour and systemic inflammatory responses characterized by increased IFN*γ*/IL-9 secreting T cells in the spleen and the tumour. Low numbers of antigen specific T cells (<20/10^6^ spleen cells) in the spleen of the tumour bearing mice are also detected after intravenous DCG delivery. Interestingly, our results showed that a mixed IL-9/IFN*γ* secreting T cell response was induced when the tumour bearing mice received a low dose of DCG spore (1 × 10^8^ CFU/kg), while a strong IFN*γ* response was elicited with a high dosage of DCG spore (3 × 10^8^ CFU/kg).

**Conclusion:**

The dosage of DCG spore will determine the types of the DCG induced immune responses.

## 1. Introduction

Cancer immunotherapy is the most promising new cancer treatment of the past years and has achieved clinical responses by enhancing the immune system to fight cancer [1, 2]. For example, blocking PD-1/PD-L1 signalling pathway by monoclonal antibodies has shown clinical responses against melanoma and other solid tumours [3–6].

Cervical cancer is the 3rd most diagnosed cancer in women worldwide [7]. It resulted from the infection of human papillomavirus, especially subtypes 16 and 18 [7]. HPV16 long E7 peptide-based vaccine has been demonstrated to be effective against vulvar intraepithelial neoplasia. However, HPV therapeutic vaccines remain to be seen as effective against cervical cancer [8, 9], probably due to the existence of the immune suppressive tumour microenvironment and the anoxia status of advanced cancer [10, 11].

Solid tumours at advanced stages show angiogenesis of abnormal blood vessels restricting blood supply and resulting in reduced oxygen levels in the tumour microenvironment [12]. As a consequence, some areas of the late stage tumour are anaerobic and necrotic [12]. Anoxia regions within the tumour limit the effectiveness of conventional and immunotherapies. However, such an environment provides a suitable condition for the proliferation of the anaerobic bacteria and an opportunity for exploring a novel method for the treatment of late solid tumours [10].

Patients with large inoperable tumours have been observed to become tumour-free after fever. Intravenous (*i.v.*) injected spores from an anaerobic bacterium germinated and proliferated inside the hypoxic environment of the solid tumour [10]. Several species of bacteria, including extracellular* Clostridium*, have been developed for bacterial-assisted oncolysis [10, 13, 14]. Numerous preclinical studies and trials have demonstrated that Derivative* Clostridial* spores (DCG) have shown distinct advantages which are selectively germinated and multiplied as rods inside solid tumours [10]. Neutrophil, Monocyte, and Lymphocyte infiltration to the tumour was observed after DCG spore treatment [15]. Administration of C.* novyi* NT to a patient with advanced leiomyosarcoma was effective at inducing tumour regression [16]. However, the levels of the cytokine and antigen specific T cell responses after intravenous administration of anaerobic bacteria treatment have not been studied thoroughly. Besides, there is a self-limiting process where anaerobic* Clostridial* spore treatment of solid tumour mass will not eradiate tumour completely, because of its anaerobic character where the peripheral rim of the tumour is often not anaerobic.

In the current paper, we aim to investigate whether* i.v.* administration of different amounts of DCG spore, which is nontoxic and nonpathogenic, results in different immune responses either systematically or tumour locally by analysing the immune cell numbers and the cytokine levels in the spleen and the tumour in a TC-1 tumour model in mice.

## 2. Materials and Methods

### 2.1. Mice

6-8-week-old adult female C57BL/6 (H-2^b^) mice were purchased specific pathogen free (SPF) from the Animal Resource Centre, Shan Dong University, and kept at the Animal Resource Centre, Shandong Xing Wei Biopharm Company, Jinan, Shandong Province, China. All experiments were approved by and performed in compliance with the guidelines of Shandong Xing Wei Biopharm Company Animal Experimentation Ethics Committee (Ethics Approval Number: JXBC20150708M).

### 2.2. Antibodies

Anti-CD45.2-FITC (104), Anti-CD8a-PerCP-Cyanine5.5 (53-6.7), Anti-CD3-FITC (17A2), Anti-CD3-APC (17A2), Anti-CD4-PE (RM4-5), Anti-NK1.1-APC (PK136), Anti-B220-PE (RA3-6B2), Anti-F4/80-PerCP-Cyanine5.5 (BM8), Anti-IFN*γ* PerCP-Cyanine5.5 (XMG1.2), Anti-IL5-PE (TRFK5), Anti-IL9-APC (RM9A4), and Anti-IL-10-PerCP-Cyanine5.5 (JES5-16E3) were purchased from eBioscience (Melbourne, Australia).

### 2.3. Cell Line

Murine TC-1 cell line was purchased from Shanghai institutes for cell resource centre, Chinese Academy of Sciences, and cultured following the protocols in the product sheets. TC-1 tumour cell line is derived from primary lung epithelial cells of C57BL/6 mice and transformed with HPV16 E6/E7, which is often used in mice model of cervical cancer [17]. Briefly, TC-1 cells were cultured in complete RPMI 1640 media supplemented with 10% heat inactivated fetal calf serum, 100 U of penicillin/ml and 100 *μ*g of streptomycin/ml (GIBCO), 0.2 mM non-essential amino acid solution, 1.0 mM sodium pyruvate, 2 mM L-glutamine, and 0.4 mg/ml G418 and were cultured at 37°C with 5% CO_2_. The TC-1 cells were routinely tested for mycoplasma as described elsewhere [18].

### 2.4. TC-1 Tumour Challenge

TC-1 tumour challenge has been described elsewhere [18]. Briefly, TC-1 cells, approximately 70% confluent, were harvested with 0.25% trypsin and washed repeatedly with PBS. TC-1 tumour cells (2 × 10^6^) were injected subcutaneously into the hind flank area in 0.2 ml of PBS using a 25G needle. TC-1 tumour bearing mice were divided into 4 different groups: (1) Untreated; (2) PBS treated; (3) Low Dose DCG treated (1x10^8^ CFU/kg); and (4) High dose DCG treated (3x10^8^ CFU/kg) group. Each group has six mice. Between 4 and 7 days later and every 3 days thereafter, the area was observed and palpated for the presence of a tumour nodule. Tumour sizes were assessed every 3 days using callipers to determine the average diameter of each tumour. Tumour volumes were calculated as width×width×length. Mice were sacrificed when the tumour diameter reached 20 mm.

### 2.5. Preparation of* C. ghonii* Spores

To prepare* C. ghonii* spores for* in vivo* anti-tumour study,* C. ghonii* spores were first retrieved from −80°C and streaked on HI medium agar plates, which were incubated at 37°C under anaerobic conditions for 2 days. Bacterial colonies were inoculated into 100 ml HI media and grown under anaerobic conditions at 37°C overnight. The bacterial culture was then added to 1 L of sporulation medium (5 g Na2HPO4, 3% peptone, 0.5 g L-cysteine, 10 g maltose, 5% w/v dried cooked meat particle (Oxoid), and pH 7.4) and incubated under anaerobic conditions at 37°C for 2 weeks for sporulation. After 2 weeks, the sporulation medium was gently mixed and centrifuged at 13000 rpm for 15 min at 4°C. The cell pellet was then washed with cold PBS twice followed by resuspending in cold PBS and sonicated for 5 min to release endospores and eradicate vegetative forms of clostridia. Spores were then washed 6-10 times with cold PBS to remove vegetative cell debris. The purity of the spore suspension was finally confirmed by microscope inspection. The spore number was determined by serial dilution and plating. To determine colony forming unit (CFU),* C. ghonii *spores were plated on HI medium agar plates and incubated anaerobically at 37°C for 2 days. CFU of on HI agar plates were counted and calculated.

### 2.6. Multicytokine ELISA for Cytokines

A multicytokine ELISA kit for cytokines was purchased from Qiagen (Australia), and experiments were performed following the instructions provided within the kit. ELISA results were read by using an ELISA plate reader (Polarstar Omega 96-well microplate reader, BMG Labtech GmBH, Germany) at 450 nm.

### 2.7. Flow Cytometric Analysis

Single spleen cells or single cells isolated from tumour were washed with wash buffer extensively with PBS containing 2% FCS, before being surface stained with relevant antibodies. After extensive wash, cells were acquired on BD FACS Calibur (Becton Dickinson). Flow cytometry data was analysed by NovoExpress™ (ACEA Biosciences, Inc., Diego, CA 92121, USA). The method has been described elsewhere [17, 18].

### 2.8. Isolation of Tumour Cells

The method has been described elsewhere [17, 18]. Briefly, tumours were excised and cut into small pieces after the removal of blood vessels and connective tissue by dissection. To isolate blood cells, tumour tissues were incubated for 1 h, with occasional shakings, in an enzyme mixture that consisted of 1 *μ*g of collagenase D/ml, 20 *μ*g of DNase I/ml, and 10% fetal calf serum in RPMI-1640 at 37°C. The digested tissue was passed through a 70 *μ*m nylon mesh, and the resultant cells were washed twice in PBS. Mononuclear cells were obtained with Lymphocyte Separation Medium (Axis shield) following a centrifugation at 2000 rpm for 25 minutes. Cells were stained for surface molecules and then fixed and permeabilized using a permeabilization kit (Biolegend) before being intracellularly stained with anti-mouse IFN-*γ*, IL-5, or IL-9, respectively, or isotype-matched control mAb for 20 minutes in the dark at room temperature. Samples were analysed by flow cytometry using a FACS Calibur analyser (Becton Dickinson).

### 2.9. ELISPOT

ELISPOT was performed as described [19]. Briefly, single spleen cells were added to a membrane base 96-well plate (Millipore, Bedford, MA) coated with anti-IFN-*γ* (BD Harlingen, San Diego, CA). HPV16 E7 MHC class I restricted peptide RAHYNIVTF was added at various concentrations and cells were held at/incubated at 37°C with peptide for 18 h. Antigen specific IFN-*γ* secreting cells were detected by sequential exposure of the plate to biotinylated anti-IFN-*γ* before* s.c.* vaccination (BD Harlingen), avidin-horseradish peroxidase (Sigma-Aldrich), and DAB (Sigma-Aldrich). The results were measured by ELISPOT reader system ELR02 (AID Autoimmun Diagnostika GmbH, Strassberg, Germany). Antigen specific CD8+ T cells were shown as the numbers of spots/10^6^ per splenic cells.

### 2.10. Statistical Analysis

Statistical analysis was performed by using the two tailed Student's test, or Mann-Whitney test using Prism 6.0 (Graphpad Software, San Diego). P<0.05 is considered statistically significant.

## 3. Results

### 3.1. Intravenous Administration of DCG Does Not Change the Numbers of T Cells in the Spleen of TC-1 Tumour Bearing Mice

We previously showed that the intravenous (*i.v.*) administration of DCG spore inhibits TC-1 growth compared with nontreated group. In the current study, the same experiment was performed and similar results were obtained (Figure S1). After* i.v.* injection of different amounts of DCG, the TC-1 growth was inhibited. The tumour growth inhibition was similar between the 1x 10^8^ and 3x 10^8^ DCG treatment.

**Figure 1:**
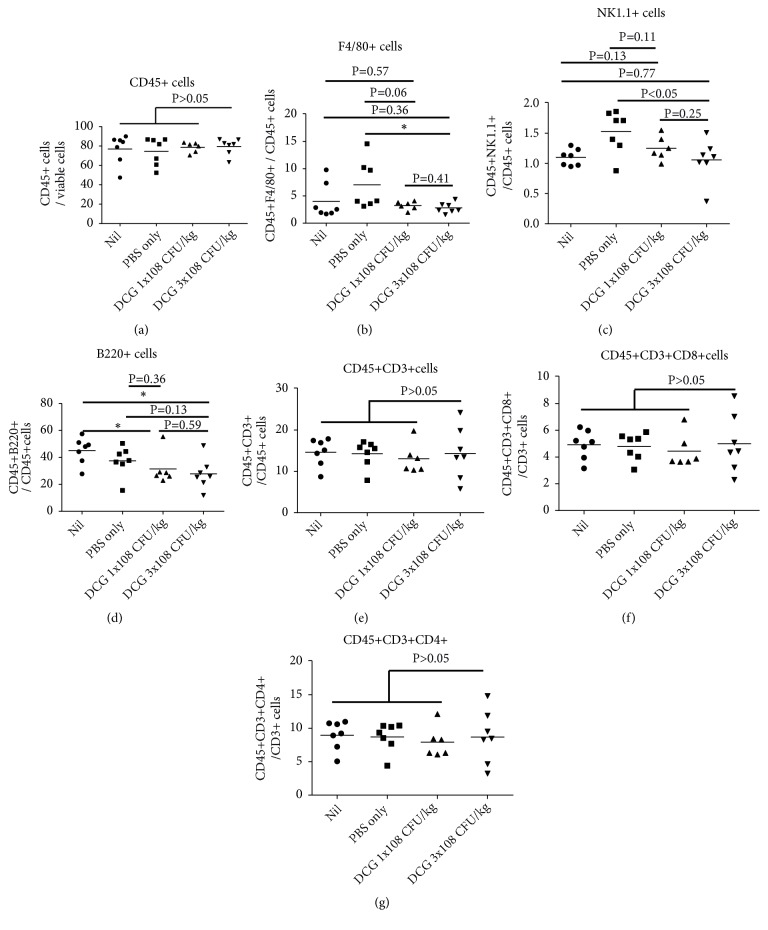
*Intravenous administration of DCG does not increase the numbers of T cells in the spleen of TC-1 tumour bearing mice*. Group of 5-7 six-week-old female C57/BL6 mice were inoculated with 2x10^6^ of TC-1 tumour cells subcutaneously. When tumour grew to 300 mm^3^ in size, tumour bearing mice were injected intravenously with either (A) 1x10^8^ CFU/kg amount of CG spores, (B) 3x10^8^ CFU/kg amount of CG spores, or (C) PBS or received (D) Nil treatment; no treatment was given to the tumour bearing mice. 11 days after CG spore injection, mice were sacrificed and spleens were isolated and single cell suspension was made by physical disruption of spleen described in Materials and Methods. Single spleen cells were stained with relevant antibodies and run through flow cytometer. Live cells were gated first; then CD45+ cells were gated. The results were analysed by NovoExpress™. (a) Numbers of CD45+ cells, (b) F4/80+; (c) NK1.1+, (d) B220+; (e) CD3+; (f) CD4+, (g) CD8+ T cells. P<0.05 is considered statistically significant. The result represents one of two independent experiments.

To investigate the immune responses elicited in the spleen of the TC-1 tumour bearing mice after the intravenous DCG spore treatment, C57/BL6 mice were inoculated with TC-1 tumour cells. When the tumour grew to 300 mm^3^ in size, tumour bearing mice were injected intravenously with either 1 × 10^8^ CFU/kg amount of DCG spores or 3 × 10^8^ CFU/kg amount of DCG spores, and nontreatment and PBS treatment were included in the control groups. Eleven days later, the percentages of hematopoietic cells of the spleens among different groups were analysed. The percentages of CD45+ cells (CD45+ T cells out of total cells) were similar among DCG treated groups and the control groups. The numbers of T cells including both CD4+ and CD8+ T cells are similar among the four experimental groups. The percentages of F4/80+, NK1.1+, and B cells of the spleen are slightly reduced in DCG spore treated groups compared with the control groups (Figure 1).

### 3.2. Intravenous Administration of DCG Spores Results in Strong IFN*γ* T Cell Responses in the Spleen of TC-1 Tumour Bearing Mice by a Low Dose of DCG Treatment

**Figure 2:**
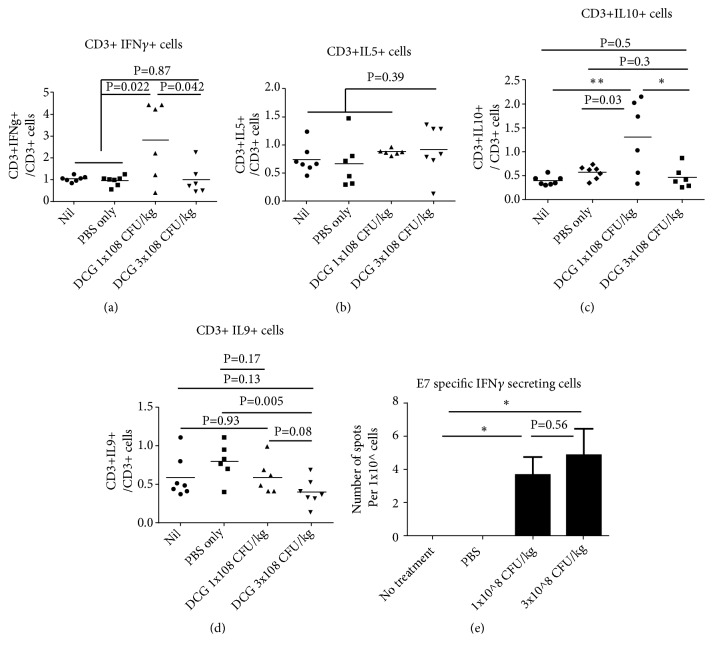
*Intravenous administration of CG results in strong Th1 T cell responses in the spleen of TC-1 tumour bearing mice*. Group of 6-7 six-week-old female C57/BL6 mice were inoculated with 2x10^6^ of TC-1 tumour cells subcutaneously. When tumour grew to 300 mm^3^ in size, tumour bearing mice were injected intravenously with either (A) 1x10^8^ CFU/kg amount of CG spores, (B) 3x10^8^ CFU/kg amount of CG spores, or (C) PBS or received (D) Nil treatment. 11 days after CG spore injection, mice were sacrificed and spleens were isolated and single cell suspension was made by physical disruption of spleen described in Materials and Methods. A total of 1x10^6^ of splenic cells were stimulated with PMA and ionomycin in the presence of protein transport inhibitor for 10 hours, and then surface was stained with anti-CD3, anti-CD4, anti-CD8a, followed by intracellular staining with anti-IFN*γ*, anti-IL-5, anti-IL-9, or anti-IL-10, respectively. Live and CD45+ T cells were gated. (a) CD3+IFN*γ*+ cells; (b) CD3+IL-5+; (c) CD3+IL-10+ T cells; (d) CD3+IL-9+; (e) or 1 × 10^6^ of splenic cells were stimulated with different amount of MHC I restricted HPV16E7 or unrelated MHC I restricted peptide overnight or left unstimulated. Each sample was added to the ELISPOT plate in triplicate. Antigen specific CD8+IFN*γ*+ T cell responses were measured by ELISPOT assay as described in the section of Materials and Methods. P<0.05 is considered statistically significant. Results shown represent one of two independent experiments.

The cytokine profile of splenic T cells was then investigated by intracellular staining. Our results showed that the percentages of IL-5 secreting T cells, which represent a Th2 response, were similar among DCG spore treated and nontreated groups; IL-9 secreting CD3+ T cells were slightly reduced in the 3 × 10^8^ CFU/kg treated group; the IL-10 secreting CD3+ T cells were significantly increased in the 1 × 10^8^ CFU/kg treated group but not in the 3 × 10^8^ CFU/kg treated group; the IFN*γ* secreting T cells were also significantly increased in the 1 × 10^8^ CFU/kg treated group but not in the 3x10^8^ CFU/kg treated group (Figure 2).

To investigate whether systematic DCG spore treatment elicits antigen specific CD8+ T cell responses in TC-1 bearing mice, HPV16 E7 specific CD8+ T cell responses were measured by an ELISPOT assay. Our results clearly showed that E7 specific CD8+ T cells were elicited after DCG treatment in CG spore treated mice, whether in 1× or 3× 10^8^ CFU/kg treated groups, although the numbers are low, less than 20 cells per million splenic cells (Figure 2).

### 3.3. Intravenous Administration of DCG Spores Leads to Strong Inflammation Responses within Tumour of TC-1 Tumour Bearing Mice

**Figure 3:**
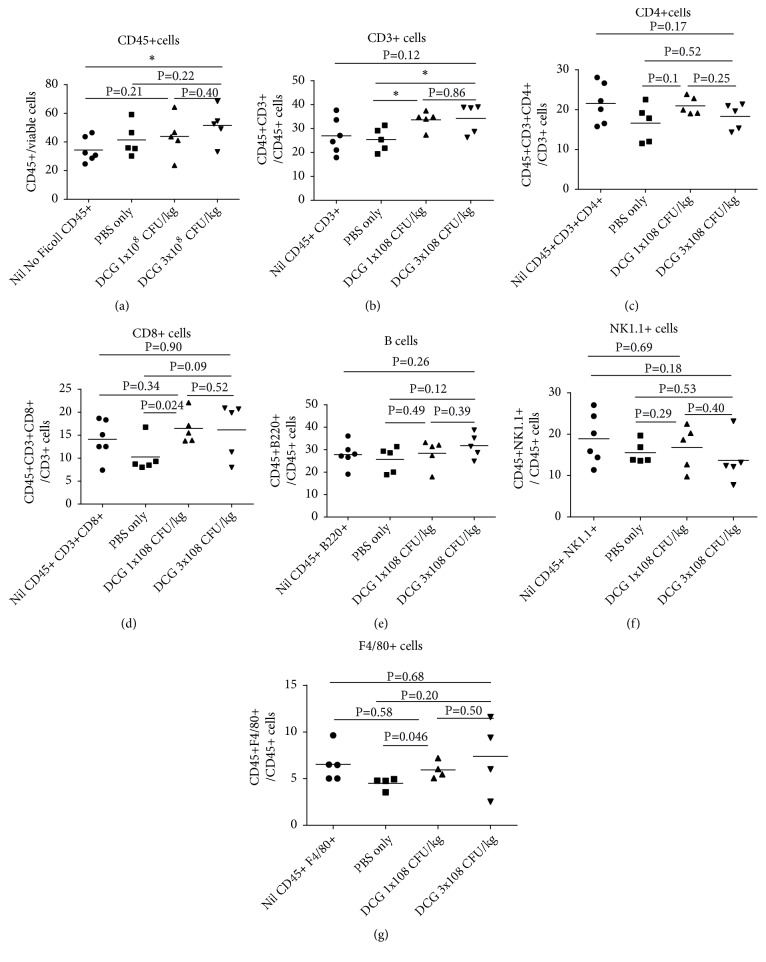
*Intravenous administration of CG does not significantly increase the numbers of T cells infiltrating to the tumour site*. Group of 5-6 six-week-old female C57/BL6 mice were inoculated with 2x10^6^ of TC-1 tumour cells subcutaneously. When tumour grew to 300 mm^3^ in size, tumour bearing mice were injected intravenously with either (A) 1x10^8^ CFU/kg amount of DCG spores, (B) 3x10^8^ CFU/kg amount of DCG spores, or (C) PBS or received (D) Nil treatment. 11 days after DCG spore injection, mice were sacrificed and tumours were isolated and single cells were made by digest with Collagenase D and cells were stained with anti-CD45. Some cells were subject to Ficoll separation to enrich mononuclear cells in tumour cells, and they were then stained with anti-CD45, anti-CD3, anti-CD4, anti-CD8a, anti-NK1.1, anti-B220, and anti-F4/80. CD45+ T cells were gated. (a) The percentages of CD45+ T cells of live cells; (b) CD3+; (c) CD3+CD4+; (d) CD3+CD8+ T cells; (e) B220+; (f) NK1.1+; (g) F4/80+ cells. P<0.05 is considered statistically significant. Results shown represent one of two independent experiments.

We then investigated the numbers of hematopoietic cells and cytokine secretion profile of T cells in the tumour after DCG spore treatment. Compared with the untreated mice, the CD45+ cells are slightly increased (without statistical significance) in DCG spore treated mice; the percentages of T cells, infiltrating to tumour, are slightly increased in DCG spore treated mice, while B220+, NK1.1+, and F4/80+ cells are similar among the DCG treated and nontreated groups (Figure 3).

**Figure 4:**
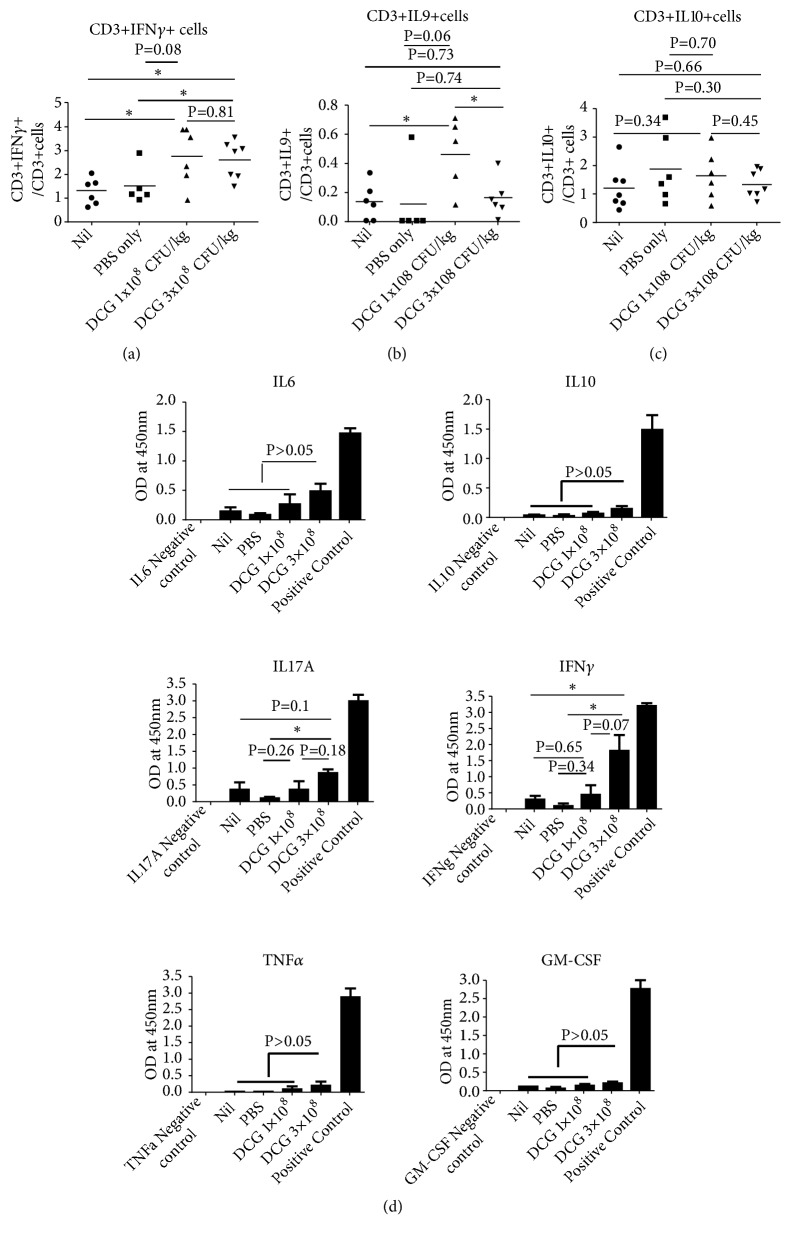
*Intravenous administration of DCG leads to strong inflammation responses within tumor of TC-1 tumour bearing mice*. Group of 5-7 six-week-old female C57/BL6 mice were inoculated with 2x10^6^ of TC-1 tumour cells subcutaneously. When tumour grew to 300 mm^3^ in size, tumour bearing mice were injected intravenously with either (A) 1x10^8^ CFU/kg amount of DCG, (B) 3x10^8^ CFU/kg amount of DCG, or (C) PBS or received (D) Nil treatment. 11 days after DCG injection, mice were sacrificed and tumours were isolated and single cells were made by digest with Collagenase D. Cells were subject to Ficoll separation to enrich mononuclear cells in tumour cells, were stimulated with PMA and ionomycin in the presence of protein transport inhibitor overnight, and were surface stained for CD45, CD3, followed by intracellular stain with anti-IFN*γ* (a), anti-IL-9 (b), and anti-IL-10 (c), respectively. Cells were gated on CD45+ cells. Or cells were stimulated with PMA and ionomycin overnight, and supernatants from stimulated mononuclear cells of the tumour were measured by multicytokine ELISA for cytokines secreted by tumour infiltrating immune cells. The multicytokine ELISA can detect IL1*α*, IL1*β*, IL4, IL5, IL6, IL10, IL13, IL17*α*, IL23, IFN*γ*, TNF*α*, and GM-CSF (d). The result represents one of two independent experiments.

The IFN*γ* secreting T cells in the 3 × 10^8^ DCG spore treated group were significantly increased compared with nontreated and PBS treated groups, while the 1 × 10^8^ DCG spore treated group is only statistically significant compared with nontreated group. Interestingly, IL-9 secreting T cells are significantly increased in the 1 × 10^8^ CFU/kg treated group compared with control groups and the 3 × 10^8^ DCG spore treated group, while IL-9 secreting T cells are similar between the 3 × 10^8^ DCG spore treated group and control groups (Figure 4).

Next, we investigated cytokine secretion profile by infiltrating T cells after nonspecific PMA/Ionomycin stimulation overnight, and the cytokines released to the supernatant were measured by multicytokine ELISA. Our results showed that the levels of IL-6, IL-17, and TNF*α* are slightly increased in DCG spore treated groups without statistical significance, but IFN*γ* is significantly increased (P<0.05) in mice treated with the 3 × 10^8^ CFU/kg DCG spore treated group, further supporting the above intracellular staining result (Figure 4).

## 4. Discussion

In the current paper, we demonstrated that intravenous administration of DCG spores to TC-1 tumour bearing mice elicited strong splenic as well as tumour local immune responses, characterised by increased IFN*γ* or/and IL-9 secreting T cells in the spleen and the tumour site. Low numbers (<20/1x10^6^ spleen cells) of tumour antigen E7 specific CD8+ T cell responses are detected in the spleen of the tumour bearing mice; moreover, whether a strong INF*γ* response or a mixed IFN*γ*/IL-9 response is determined by the dosage of DCG spore administered intravenously to the TC-1 tumour bearing mice.

It has been shown previously that facultative anaerobic bacteria colonizing solid tumors often result in tumor growth retardation or even clearance. This phenomenon has been observed in tumour models of mouse, rat, and dog and in clinics [16, 20–23]. When* C. novyi-NT *spores were administered together with conventional chemotherapeutic drugs, extensive hemorrhagic necrosis of tumour often developed within 24 hours, resulting in significant and prolonged antitumour effects [24]. The bacteria were also found to markedly improve the efficacy of radiotherapy in several mouse models tested [25]. Innate and adaptive immune mediated responses have been shown to play a critical role for the antitumour effect of anaerobic bacteria treatment. Rechallenging mice which had cleared tumour showed that clearance was due to a specific immune reaction. The therapeutic effects of anaerobic bacteria treatment are reduced in TLR4-/- mice [26]. Depletion experiments revealed that, during induction phase, CD8+ T cells are the sole effectors responsible for tumour clearance while in the memory phase CD8+ and CD4+T cells were involved. Detailed analyses of adoptively transferred CD4+ T cells during tumor challenge revealed the expression of granzyme B, FasL, TNF-*α*, and IFN-*γ* in such T cells that might be involved in the antitumour activity [27]. In another study, it was found that approximately 30% of mice treated with anaerobic bacteria were cured of their cancers despite the viable tumor rim initially remaining after spore germination. The mechanism underlying this effect was shown to be immune-mediated, because cured animals rejected a subsequent challenge of the same tumour. Similar effects were observed in rabbits with an intrahepatic tumour. It was particularly notable that the induced immune response, when combined with the bacteriolytic effects of* C. novyi-NT*, could eradicate large established tumours [15].

We further demonstrated in the current study that the DCG administration results in increased levels of IFN*γ*/IL-10 in the spleen and increased IFN*γ*/IL-9 secreting T cells infiltrating to the tumour site. High dose (3 × 10^8^ CFU/kg) of DCG spore treatment leads to increased IFN*γ* secreting T cells in tumour, and low dose (1 × 10^8^ CFU/kg) of DCG spore treatment leads to both IL-9 secreting T cells infiltrating to the tumour site, while the numbers of splenic IL-10 secreting T cells are increased in the low dose DCG spore treated group. IL-9 secreting T cells have been shown [28] to mediate antitumour immunity in a melanoma model. It is not yet to know whether the IL-9 secreting T cells in low dose DCG spore treated mice are contributing the antitumour effect, as IFN*γ* has been shown to be critical for rejecting the tumour. It seems that the mechanism underlying the tumour growth inhibition in low and high dose DCG treated mice is different, as more IFN*γ* secreting T cells were infiltrated to the tumour site in high dose DCG treated mice, while more IL-9 secreting T cells were observed in low dose DCG treated mice.

E7 specific IFN*γ* secreting CD8+ T cells from the spleens of DCG treated mice were also detected, although at low numbers. E7 specific IFN*γ* secreting CD8+ T cells cannot be detected from the tumour by ELISPOT assay. Antigen specific CD8+ T cells that infiltrate to the tumour site are critical for killing the tumour cells; therefore, attempts should be made to increase the antitumour T cell responses mediated by anaerobic bacteria treatment. Genetically modified anaerobic bacteria that express HPV16 E7 may be useful to increase the E7 specific CD8+ T cell responses.

Taken together, the DCG spore treatment increases the levels of proinflammatory cytokines at the tumour site and attracts IFN*γ*/IL-9 secreting T cells to the tumour. In future, DCG spore treatment should induce and attract more tumour antigen specific T cells to the tumour site; the new strategy warrants a further investigation.

## Data Availability

The data used to support the findings of this study are available from the corresponding author upon request. Supplementary data is available as a separate file and is included among the article's files.

## Abbreviations

HPV: Human papillomavirus
CTL: Cytotoxic T lymphocyte
DCG: Derivative of Clostridial ghonii
IFN: Interferon.

## Supplementary Materials

Supplementary Materials Figure S1: intravenous administration of DCG inhibits tumour growth of TC-1 tumour bearing mice. Group of 5-7 six-week-old female C57/BL6 mice were inoculated with 2×10^5^ of TC-1 tumour cells subcutaneously. When tumour grew to 300mm^3^ in size, tumour bearing mice were injected intravenously with either (a) 1×10^8^ CFU/kg amount of DCG, (b) 3×10^8^ CFU/kg amount of DCG, or (c) PBS or received (d) Nil treatment. 11 days after DCG injection, mice were sacrificed, tumour was isolated, and tumour weight was measured. Results represent pooled an independent experiment of tumour weight percent change.
