# Supplementary material for: The Dosage of the Derivative of Clostridium Ghonii (DCG) Spores Dictates Whether an IFNγ/IL-9 or a Strong IFNγ Response Is Elicited in TC-1 Tumour Bearing Mice
Source: Biomed Res Int. 2019 Apr 28;2019:1395138. doi: 10.1155/2019/1395138 (PMC6512072; doi:10.1155/2019/1395138)

**Figure S1. Intravenous administration of DCG inhibit tumour growth of TC-1 tumour bearing mice**

Group of 5-7 six-week-old female C57/BL6 mice were inoculated with 2×10^5^ of TC-1 tumour cells subcutaneously. When tumour grew to 300mm^3^ in size, tumour bearing mice were either injected intravenously with **a:** 1×10^8^CFU/kg amount of DCG, **b:** 3×10^8^CFU/kg amount of DCG, **c:** PBS or **d:** Nil treatment. 11 days after DCG injection, mice were sacrificed and tumour isolated, tumour weight was measured. Results represent pooled an independent experiment of tumour weight percent change.


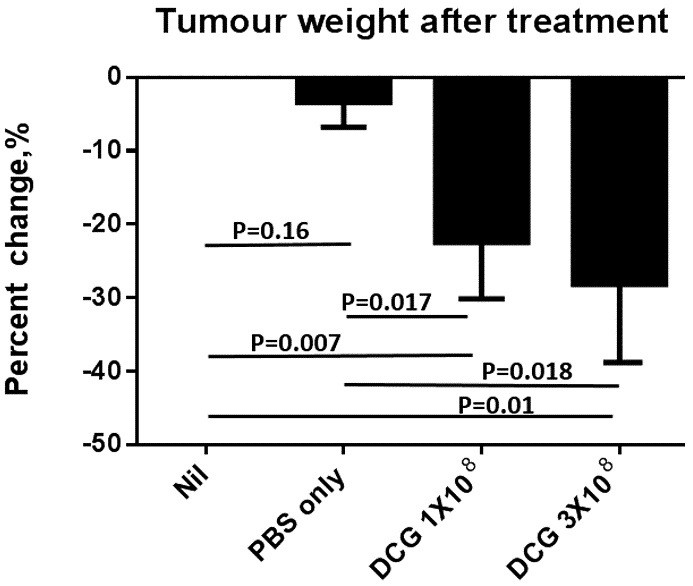

Supplement: Supplementary Materials — Figure S1: intravenous administration of DCG inhibits tumour growth of TC-1 tumour bearing mice. Group of 5-7 six-week-old female C57/BL6 mice were inoculated with 2×10^5^ of TC-1 tumour cells subcutaneously. When tumour grew to 300mm3 in size, tumour bearing mice were injected intravenously with either (a) 1×10^8^ CFU/kg amount of DCG, (b) 3×10^8^ CFU/kg amount of DCG, or (c) PBS or received (d) Nil treatment. 11 days after DCG injection, mice were sacrificed, tumour was isolated, and tumour weight was measured. Results represent pooled an independent experiment of tumour weight percent change. [file 1395138.f1.docx]
